# Joint Expedition: Exploring Telehealth and the Digital Healthcare Landscape as a Team Integration

**DOI:** 10.3390/healthcare12050585

**Published:** 2024-03-04

**Authors:** Daniele Giansanti

**Affiliations:** Centre TISP, Istituto Superiore di Sanità, 00166 Rome, Italy; daniele.giansanti@iss.it

## 1. The Joint Expedition Exploring Telehealth and the Digital Healthcare Landscape

The TeleHealth and digital healthcare domains have witnessed remarkable advancements in recent years (Contribution 1), propelled by the evolution of digitization processes, both in mobile [1,2] and in fixed technologies [3,4]. Additionally, the standardization and incorporation of the developments within the health domain [5,6] have played a crucial role in facilitating these advancements. A significant impulse in this field was the pandemic, which was a significant catalyst, providing an unprecedented growth stimulus and acting as a testing ground for the consolidation of existing areas and exploration of new applications [7,8].

Presently, the dominions of TeleHealth and digital healthcare leverage emerging technologies such as robotics [9], virtual reality [10], augmented reality [11], and artificial intelligence [12]. Even assistive technologies employed in various applications find ample opportunities within this sector [13]. In light of these considerations, we introduced a Special Issue entitled The 10th Anniversary of *Healthcare*—TeleHealth and Digital Healthcare https://www.mdpi.com/journal/healthcare/special_issues/4R7KYJ9CAJ (accessed on 20 February 2024) to coincide with the 10th anniversary of *Healthcare*. The objective was to comprehensively outline the ongoing developments, share established experiences, explore future prospects, and highlight persisting challenges in this dynamic field.

**Figure 1:**
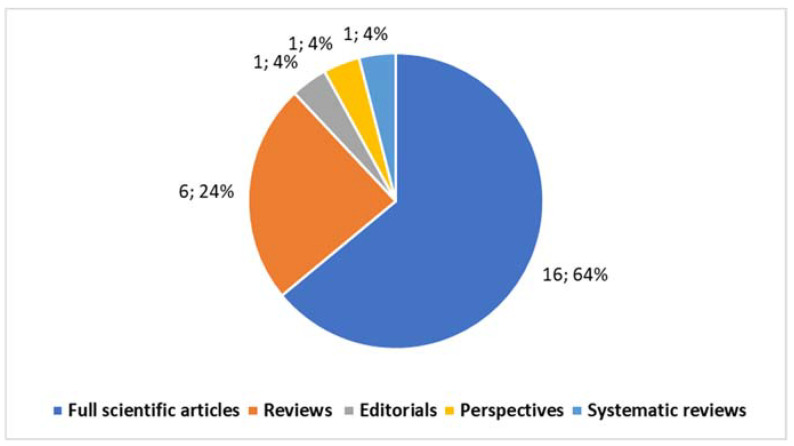
Published articles categorized.

The Special Issue has successfully achieved a significant milestone, featuring 25 contributions (excluding this editorial) (Contribution 1–Contribution 25), attracting the attention of 26,701 authors at the time of writing. The published papers, according to the selected categories (Figure 1), encompass 1 introductory editorial (Contribution 1), 16 full scientific articles (Contribution 2–Contribution 17), 6 reviews (Contribution 18–Contribution 23), 1 perspective (Contribution 24), and 1 systematic review (Contribution 25).

The Editorial by Giansanti D. (Contribution 1) introduced the aims of the SI and reflected on the progress and status of telehealth and digital healthcare over the past decade. The focus was on assessing the current state of telehealth and digital healthcare and exploring their evolution over the past ten years. It covered the historical context, technological advancements, adoption rates, patient outcomes, challenges, and future prospects in these fields.

## 2. Conclusive Discoveries: A Closer Look at Scientific Article Outcomes

### 2.1. An Overview of the Contributions

The 16 articles covered various topics of interest to the SI. Below, the focus and a brief excerpt of the content are detailed.

Ouédraogo et al.’s work (Contribution 2)—*Acceptability of Telerehabilitation*:

This study investigated the acceptability of telerehabilitation (TR) among individuals with stroke and their caregivers. Employing a qualitative approach and the UTAUT-2 model, the research revealed positive experiences with TR, showcasing improvements in functional abilities. Actors considered the technology easy to use, with prior experiences and support playing crucial roles in acceptance. On the one hand, the pandemic further acted as a catalyst in this field. On the other hand, technology, Internet instability, and lack of feedback emerged as barriers. Despite these challenges, the study emphasizes TR’s potential for effective stroke rehabilitation, urging the need to address obstacles to widespread adoption.

Shalom et al.’s work (Contribution 3)—*Evaluation of Telephone Visits in Primary Care*:

As telehealth, particularly telephone visits, becomes increasingly prevalent, this study explored family physicians’ and pediatricians’ perceptions of three key aspects: quality of care, safety, and satisfaction. A survey involving 342 participants was conducted, along with subsequent in-depth interviews. The outcome of the involved physicians highlighted a high acceptance/satisfaction with the telephone-based visits. The parameters related to the safety and the quality of the procedure received a comparatively lower assessment. Notably, 80% recommended combining face-to-face and telephone visits, and 51% acknowledged challenges in decision-making due to the inability to closely examine patients. The study emphasizes the importance of careful implementation, patient selection, technological upgrades, and patient education to enhance the effectiveness of telephone visits in healthcare.

Stara et al.’s work (Contribution 4)—*The Impact of a Multicomponent Platform Intervention on Older Adults*:

This study delves into the realm of gerontechnology, a cross-disciplinary field merging gerontology and technology to address age-related physical and cognitive decline in older adults. Focused on a multicomponent platform integrating ambient sensors, wearable devices, and a cloud application, the research evaluates its impact on self-efficacy, acceptance, well-being, and quality of life in a sample of elderly people. Utilizing a mixed-methods approach, the study indicates positive participant engagement with the integrated platform. Results highlight favorable usability and acceptance, particularly for the smartwatch, with a slight improvement in overall well-being and stable self-efficacy. The study underscores the readiness of such technology for mainstream use, offering valuable insights for developers targeting the aging population.

Rossi et al.’ s work (Contribution 5)—*Social Media Perspectives on the Inspire Upper Airway Stimulation System*:

This study delves into the landscape of sleep surgery, focusing on the Inspire^®^ Upper Airway Stimulation (UAS) device, a popular treatment for obstructive sleep apnea, and its representation on social media platforms (Instagram, Facebook, TikTok). Analyzing 423 public posts, the study identifies images (67.4%) and videos (28.1%) as dominant content, with physicians contributing over a third of the posts. Notably, 40% are advertisements, and patient experiences constitute 34.5%. TikTok exhibits higher engagement, with posts averaging 152.9 likes, compared to Instagram and Facebook at 32.7 and 41.2 likes, respectively. The findings emphasize the importance of healthcare professionals providing clear, evidence-based information in the evolving landscape of healthcare on digital platforms. Collaborative efforts are crucial to ensure accurate health information dissemination in the realm of medical innovations like the UAS device.

Litvinova et al.’s work (Contribution 6)—*Patent and Bibliometric Analysis of Pulse Oximeters in Digital Medicine*:

This study comprehensively examined pulse oximeters’ evolution in digital medicine through patent and bibliometric analysis. Key countries dominating pulse oximeter patents include the United States, China, the Republic of Korea, Japan, Canada, Australia, Taiwan, and the United Kingdom. The analysis highlighted a consistent growth trend, underscoring the increasing significance of pulse oximeters in digital medical practices. Utilizing a specific software, six primary research clusters were identified, spanning measurement accuracy, IoT integration, diverse pathology applicability, telemedicine, AI and deep learning, and usage in critical care settings. The findings suggest promising applications of digital technologies in pulse oximetry across various medical fields, with identified technical solutions to enhance accuracy, validity, and clinical use and incorporate machine learning.

Giannopoulou et al.’s work (Contribution 7)—*RODI mHealth app Insight for Neurodegenerative Disorder Detection*:

This study focuses on addressing the global health concern of Neurocognitive Disorders (NCDs) by developing the RODI mHealth app. Conducted from July to October 2022 with 182 participants (both with NCDs and healthy individuals), the study aims to assess performance differences, identify critical features for outcome prediction, and utilize machine learning to unveil patterns associated with NCDs. The analysis prioritizes tasks within RODI based on their alignment with NCD criteria, revealing that tasks related to visual working memory are most significant in distinguishing between healthy individuals and those with an NCD. The study provides a blueprint for future mHealth apps, guiding the enhancement of digital indicators for disorders and related conditions.

Mustapoevich and Kim’s work (Contribution 8)—*Machine Learning Applications in Sarcopenia Detection*:

This study focuses on sarcopenia, a condition characterized by muscle mass loss, stamina decline, and reduced physical performance, with a specific emphasis on its detection and management using modern technologies. The review explores the lack of global consensus on sarcopenia’s definition and the diverse techniques for measuring its parameters. It discusses criteria from the European and Asian Working Groups, highlighting challenges in result comparison. The paper delves into machine learning applications for sarcopenia detection, emphasizing issues like data accessibility and feature selection. Wearable devices, blockchain technology, and edge computing are also explored for monitoring, data security, and healthcare information management. While recognizing potential benefits, the review underscores limitations and calls for further research to address standardization and data management challenges and optimize technology use in sarcopenia detection and management.

Gallè et al.’s work (Contribution 9)—*Introducing Telemedicine in Italy: Citizens’ Awareness*:

This study investigates the awareness and attitudes toward telemedicine in the Italian adult population, particularly considering sociodemographic factors and regional differences in service implementation. Conducted between October 2022 and February 2023, the questionnaire-based research reveals that less than half of the respondents were aware of telemedicine services in their region. Among those who were aware, limited usage was attributed to preferences for in-person visits or a perceived lack of need. Over 90% of users expressed satisfaction with telemedicine services. Notably, a negative attitude toward telemedicine was more prevalent among older adults. The study emphasizes the need for increased awareness and the utilization of telemedicine in Italy despite its active presence.

Pennestrì and Banfi’s work (Contribution 10)—*Primary Care improvements*:

The contribution starts highlighting the importance of the allocation of European funds in the Italian digitalization processes related to the health domain. While national guidelines emphasize telemedicine as a key innovation, this paper delves into the specific contributions that digital health and telehealth can play in primary healthcare modernizations. The study explores how digital solutions can support effective stratification, prevention, and management of chronic patient needs. It addresses key areas involving population health management, chronic care management, clinical groups, anticipatory healthcare, quality and outcome frameworks, patient-reported outcomes, and patient-reported experience. The paper highlights the potential benefits of digitalization while acknowledging the associated risks and limitations that need to be considered in the implementation process.

Cho and Hong’s work (Contribution 11)—*Electronic Sharing of Health Information and Costs*:

This study explores the impact of the electronic sharing of health information on hospital costs and identifies circumstances leading to changes in overall hospital performance. Utilizing data from sources including the American Hospital Association, Center for Medicare and Medicaid Services, and Census Bureau, the research finds a considerable decrease in hospitalization costs by sharing the information internally but not outside. Interestingly, the study reveals that despite challenges such as lack of incentives and capabilities, sharing health information can still lead to cost savings for hospitals. The implication suggests that overcoming obstacles in information sharing is crucial for hospitals to realize the anticipated advantages. The study recommends policymakers target hospitals facing challenges in information sharing, emphasizing a need for strategic approaches to address specific obstacles and improve policy efficiency.

Yang et al.’s work (Contribution 12)—*Kinesio Taping and Virtual-Reality-Based Upper Extremity Training for Stroke Patients*:

This study investigates the impact of combining hand motion training based on virtual reality using the Kinesio Taping (KT) system on stroke. Forty-three stroke patients were randomly assigned to either an experimental group receiving both KT and VRT or a control group receiving only VRT over 30 sessions spanning 6 weeks. Evaluation tools included the Wolf Motor Function Test (WMFT), the Fugl–Meyer Assessment of the Upper Extremity (FMA-UE), the Motor Activity Log (MAL), and the Self-Efficacy Scale (SEF). The experimental group demonstrated statistically significant improvement in FMA-UE, WMFT, MAL, and SEF compared to the control group. The study concludes that the combined intervention of VRT with the KT technique positively impacted the upper limb recovery in the involved patients, suggesting that the stability provided by KT to the wrist extensor muscles contributed to more effective improvement than VRT alone.

Vintilă et al.’s work (Contribution 13)—*Digital Healthcare Communication for Urologists’ Surveillance of Lithiasis Patients*:

This paper addresses the impact of the COVID-19 pandemic on lithiasis patients, particularly those requiring internal stents. Two studies were conducted: a clinical study evaluating the bacterial urinary colonization prevalence in patients with obstructive urolithiasis and internal stents and a quantitative study using multiple linear regression to gauge urologists’ opinions on leveraging digital technologies for improved communication. The clinical study found a 35% prevalence of urinary colonization, affected by infection in combination with the SARS-CoV2 virus. The outcome indicated urologists’ openness to employing online technologies for communication with patients. The results provide valuable insights for healthcare practitioners, emphasizing factors influencing the communication process and suggesting considerations for hospital managers when implementing online communication technologies.

Gatica et al.’s work (Contribution 14)—*Teledermatology Evaluation and Feedback Systems*:

This study evaluates Chile’s teledermatology system by analyzing 243 consultations. It focuses on basic specifiers’ fulfillment, revealing strong adherence to core teledermatology functions. Significant correlations are found between patient destination, pharmacological prescription, drug coverage, and physician education. Primary health center consultations exhibit higher pharmacological prescription rates, mostly involving government-covered drugs, compared to face-to-face referrals. The study emphasizes targeted evaluations for education and prescriptions to enhance teledermatology quality.

Niculescu et al.’s work (Contribution 15)—*Challenges of Integrating New Technologies for Orthopedic Doctors*:

This research explores the intention of orthopedic doctors to adopt new medical technologies, particularly during the pandemic era. The study, encompassing 145 orthopedic doctors, utilized a questionnaire for data collection and employed a multiple linear regression model for analysis. The findings highlight that doctors’ intention to embrace new medical technologies is influenced by perceived advantages and disadvantages, risks, technology quality, experience in usage, and receptivity to digital tools. The results provide valuable insights for hospital managers and authorities, shedding light on key factors influencing the adoption of emerging technologies in orthopedics.

AlAli et al.’s work (Contribution 16)—*Usage of Digital Health Mobile-Based Applications among Saudi Population*:

This cross-sectional study investigates the adoption of digital health mobile applications among the Saudi population to guide the Saudi Ministry of Health and the government in scaling up digital health initiatives. The research explores the extent of usage, affordability of smart devices, app presence, perceived benefits, and barriers to adoption. Findings reveal that while many participants can afford smart devices and recognize the benefits of medical apps, limited understanding of usage, difficulties in downloading, and ethical concerns hinder widespread acceptance. The study emphasizes the need for additional efforts from authorities to promote the uptake of digital health in Saudi Arabia.

David et al.’ work (Contribution 17)—*Mobile Health Interventions on Lifestyle and Anthropometric Characteristics*:

This randomized controlled trial aimed to evaluate the effect of mHealth procedures on subjects with hypertension without control. Participants received lifestyle counseling and were randomly assigned to different mHealth interventions or usual clinical treatment (control). The mHealth group, which included an automatic oscillometric device for blood pressure measurement and personalized text messages, showed a significantly higher likelihood of achieving lifestyle goals at six months. Additionally, there were clinically relevant reductions in body fat, segmental trunk fat, and waist circumference in the mHealth group compared to the control, emphasizing the positive impact of mHealth on hypertension management and lifestyle adherence.

### 2.2. Common Message

The combination of digital technologies and healthcare has ushered in transformative advancements, as evidenced by a comprehensive exploration of 16 scientific article studies (Contribution 2–17). From the widespread acceptance of Telehealth interventions (Contribution 2–3) to the impactful integration of multicomponent platforms for older adults (Contribution 4), the studies underscore a paradigm shift in healthcare delivery. Insights from social media perspectives (Contribution 5) emphasize the need for accurate information dissemination, reflecting the evolving landscape of healthcare on digital platforms.

Technological innovations, such as machine learning applications (Contribution 8), mHealth apps for neurodegenerative disorder detection (Contribution 7), and pulse oximeter evolution in digital medicine (Contribution 6), reveal promising prospects for the future. The studies collectively highlight the potential benefits of digital technologies in addressing healthcare challenges.

Public awareness and acceptance of telemedicine, as explored in Italy (Contribution 9), and the forward-looking approach to primary care innovations in Italy (Contribution 10), underscore the critical role of technology-driven policies in shaping healthcare. The exploration of the electronic sharing of health information (Contribution 11) provides strategic insights for policymakers, emphasizing the importance of overcoming obstacles for hospitals to realize anticipated advantages. Innovative healthcare approaches in diverse regions, such as Saudi Arabia (Contribution 16), showcase the potential for digital health adoption while acknowledging challenges. The impact of mobile health interventions on lifestyle and anthropometric characteristics (Contribution 17) points to the positive influence of digital solutions on hypertension management.

Studies delving into specialized healthcare domains, such as the combined intervention for stroke patients (Contribution 12), digital communication in urologists’ surveillance (Contribution 13), and teledermatology evaluation (Contribution 14), reveal targeted applications of technology in critical medical areas.

### 2.3. Key Emerging Themes

As a whole, these studies (Contribution 2–17) reflect a dynamic landscape where technology, carefully implemented, holds the promise to enhance healthcare accessibility, quality, and patient outcomes. The collective findings call for collaborative efforts among healthcare professionals, policymakers, and technologists to navigate challenges and leverage the full potential of digital healthcare innovations (Contribution 2–17). The articles embraced various themes, with each article encompassing different ones. Focusing on the dominant theme, Table 1 illustrates the key emerging dominant themes along with the corresponding articles.

## 3. Conclusive Discoveries: A Closer Look at the Reviews and the Perspective

### 3.1. An Overview of the Contributions

The seven reviews, including the systematic reviews, provide a significant contribution to the exploration of more stabilized emerging themes proposed in the collection (Contribution 18–23,25). The perspective included in this collection helps us look toward the future (Contribution 24) of this discipline. Below, the focus and a brief excerpt of the content are detailed.

Holl et al.’s work (Contribution 18)—*Mobile Apps for COVID-19: A review of Reviews*.

This study conducts an overview of mobile applications (apps) used during the COVID-19 pandemic. Analyzing 24 eligible studies, the review covers the period from January 2020 to April 2022. Key findings highlight that most reviews focused on apps from the USA, the UK, and India, leaving a gap regarding apps from many African and Middle and South American countries. The categorization reveals four main groups: security and privacy, App overview, MARS rating, and miscellaneous. The study aims to offer a high-level overview, identify factors contributing to app success, and pinpoint gaps in the current literature, providing valuable data for future analyses and research.

Alghamdi’s work (Contribution 19)—*Effective Telehealth Solutions for COPD: A Narrative Review*.

This paper explores Telehealth (TH) solutions as promising interventions for managing Chronic Obstructive Pulmonary Disease (COPD). Conducting a literature review up to October 2023, the study identifies 30 papers presenting TH solutions for COPD management. TH and digital health solutions are considered interchangeable, both aiming to enhance care, accessibility, and quality of life. The content of TH solutions encompasses symptom management, physical activity promotion, and psychological support. Mechanisms are influenced by factors like content, delivery mode, strategy, and intensity. Common outcomes include treatment adherence, health status, and quality of life. Effective implementation requires consideration of patient needs, technology familiarity, healthcare professional support, and data privacy, emphasizing patient engagement for optimal effectiveness.

Simeoni al.’s work (Contribution 20)—*Assistive Tech Impact in International Web Portals*.

This study explores the role of assistive technologies (ATs) in supporting individuals with disabilities and frailties, focusing on the initiatives of the UN, UNICEF, and WHO. Using a dual-method approach, the research involves a direct search of institutional websites and a literature review. The findings reveal initiatives aimed at tailoring ATs based on guidelines, monitoring their introduction through surveys, and disseminating AT culture and recommendations. The study indicates a growing interest in ATs, with international institutions playing a vital role in monitoring, disseminating, and improving access to ATs. However, persistent challenges need to be addressed to enhance the success of these initiatives.

Almufareh et al.’s work (Contribution 21)—*Motor Imagery Rehabilitation for Individuals with Disabilities: A Review*.

This manuscript provides a comprehensive analysis of the significance of motor imagery in neuro-rehabilitation, emphasizing its non-invasive and cost-effective nature. It explores the fundamental mechanisms, applications across various disability conditions, and potential benefits for enhancing motor functionality. The document also discusses existing challenges and highlights the need for ongoing research and innovative technologies to maximize the potential of motor imagery in aiding individuals with disabilities.

Borna et al.’s work (Contribution 22)—*A review on AI Models in Health Information Exchange*.

This review explores the utilization of AI models in Health Information Exchange (HIE) to predict clinical outcomes, addressing a gap in current research. The study, following PRISMA guidelines, analyzed 11 shortlisted publications out of 1021 identified through databases. The findings reveal a notable preference for machine learning models, particularly in oncology and cardiac failures, showcasing commendable predictive proficiency with varying metrics. While AI in HIE holds transformative potential for healthcare, the study emphasizes the need for a well-rounded approach to ensure the delivery of trustworthy and effective AI-augmented healthcare solutions.

Morone et al.’s work (Contribution 23)—*Assistive Technologies in Spinal Cord Injury: A Narrative Review*.

This narrative review of reviews explores the integration of assistive technologies (ATs) in the context of Spinal Cord Injury (SCI). Conducted through a search on PubMed and Scopus, the review identifies the evolution of ATs, emphasizing their role as products, services, or standalone or networked devices. It underscores the potential of innovative technologies to enhance the quality of life and reduce healthcare costs in SCI. However, the review identifies gaps in addressing ethical and regulatory aspects, emphasizing the need for comprehensive studies focusing on multiple domains to facilitate integration into the health domain.

Calvache-Mateo et al.’ work (Contribution 25)—*Respiratory Telerehabilitation in Long COVID-19: A Systematic Review*.

This systematic review and meta-analysis focused on exploring the use of telerehabilitation for supporting Long COVID-19 rehabilitation. The review included controlled trials comparing respiratory telerehabilitation interventions with various controls. The analysis, encompassing 10 studies, revealed significant positive outcomes for telerehabilitation in improving quality of life, reducing dyspnea, and enhancing strength both in respiratory muscles and lower limb muscles while improving the functional capacities of the involved subjects. The study did not show a significant difference in adverse outcomes between comparator groups and telerehabilitation groups. The findings highlighted both the effectiveness and safety of the methodology for Long COVID-19 patients.

Cruz-Panesso et al.’s work (Contribution 24)—*Training Physicians for Telehealth Competencies*.

This perspective study highlights the increasing prevalence of telehealth in North America, emphasizing a 40% rise between 2019 and 2020, stabilizing at 40% in 2021. It underscores the challenges in integrating telehealth curricula due to a shortage of experienced faculty and limited time. To address these issues, the article recommends rethinking traditional learning models and suggests telesimulation as an effective method for training telehealth competencies, providing practical experiences for technical and interpersonal skills.

### 3.2. Common Message

Across diverse healthcare reviews, a unified message resonates—the urgent need for ongoing innovation, adaptive strategies, and a patient-centric paradigm within the evolving healthcare landscape (Contribution 18–25). The discourse spans specific domains like mobile apps, telehealth solutions, assistive technologies, motor imagery rehabilitation, AI in health information exchange, and respiratory telerehabilitation. The emerging focus is on:

Innovation and Patient-Centricity:

The reviews and the perspective collectively advocate for leveraging cutting-edge technologies (Contribution 18–23, 25), including mobile apps, telehealth solutions, AI models, and assistive technologies, to address healthcare challenges. The common thread emphasizes the importance of tailoring interventions to meet individual patient needs, fostering engagement and adherence, and considering diverse requirements.

Global Perspective and Challenges:

Certain reviews offer a global outlook, stressing the role of international institutions and the challenges in disseminating assistive technologies globally (Contribution 20, 23). Identifying existing challenges and gaps, whether in the analysis of mobile apps from specific regions or integrating assistive technologies ethically, is a recurring theme.

Efficacy, Safety, and Evidence-Based Practices:

Systematic evaluations, such as respiratory telerehabilitation in Long COVID-19, consistently underscore the efficacy and safety of interventions (Contribution 24). The common discourse prioritizes evidence-based practices, positive patient outcomes, and the integration of proven strategies into healthcare protocols.

Call for Ongoing Research:

All the contributions (Contribution 18–25) advocate for sustained research and development. Whether in motor imagery rehabilitation, AI models, or telehealth competencies, there is a shared call for continuous exploration, improvement, and adaptation to emerging healthcare needs.

### 3.3. Key Emerging Themes

In essence, the collective discourse revolves around embracing innovation, prioritizing patient well-being, acknowledging global perspectives and challenges, ensuring efficacy and safety, and advocating for continuous research and improvement within the dynamic healthcare landscape.

The contributions embraced various themes, with each one encompassing different themes. Focusing on the dominant theme, Table 2 illustrates the key emerging dominant themes along with the corresponding study.

### 3.4. In Conclusions

Recent technological innovations, including AI, and the COVID-19 pandemic have spurred a formidable acceleration in research and development within digital health, significantly propelling mHealth and eHealth. The Special Issue gathered important contributions in various domains, identifying both emerging and well-established themes and outlining intriguing directions for future developments. The initiative also underscores the significance of these tools as a focal point for scholarly exchange and discussion among researchers worldwide.

## Figures and Tables

**Table 1:** Dominant emerging theme by article.

Themes	Description	Studies
*Telehealth and Digital Interventions*	Acceptability of Telerehabilitation. Evaluation of Telephone Visits in Primary Care	(Contribution 2) (Contribution 3)
*Impact of Digital Platforms on Healthcare*	Impact of a Multicomponent Platform on Older Adults Social Media Perspectives on Healthcare Devices	(Contribution 4) (Contribution 5)
*Technological Advancements in Medicine*	Patent and Bibliometric Analysis of Pulse Oximeters mHealth App Insight for Neurodegenerative Disorders Machine Learning Applications in Sarcopenia Detection	(Contribution 6) (Contribution 7) (Contribution 8)
*Telemedicine and Public Awareness*	Introducing Telemedicine in Italy: Citizens’ Awareness Primary Care of the (Near) Future	(Contribution 9) (Contribution 10)
*Specialized Healthcare Domains*	Combining Virtual-Reality-Based Training for Stroke Patients Digital Healthcare Communication for Urologists’ Surveillance Teledermatology Evaluation and Feedback Systems	(Contribution 12) (Contribution 13 (Contribution 14)
*Innovative Healthcare Approaches*	mHealth Interventions on Lifestyle and Anthropometric Characteristics Usage of Digital Health Mobile-Based Applications in Saudi Arabia	(Contribution 17) (Contribution 16)
*Health Information Sharing and Technology Adoption*	Various Medical Technologies Adoption by Orthopedic Doctors Electronic Sharing of Health Information and Costs	(Contribution 15) (Contribution 11)

**Table 2:** Dominant emerging theme by study.

Themes	Description	Studies
*Assistive Technology*	Assistive Tech Impact in International Web Portals Assistive Technologies in Spinal Cord Injury Integration	(Contribution 20) (Contribution 23)
*Physician training in digital health*	Training Physicians for Telehealth Competencies: The perspective in North American territory	(Contribution 24)
*Rehabilitation and digital health*	Respiratory Telerehabilitation in Long COVID-19: A Systematic Review Motor Imagery Rehabilitation for Individuals with Disabilities: A Comprehensive Review	(Contribution 25) (Contribution 23)
*AI Integration*	AI Models in Health Information Exchange: Clinical Implications	(Contribution 22)
*Effectiveness of telehealth* *solutions*	Effective Telehealth Solutions for COPD: A Narrative Review	(Contribution 19)
*Mobile Apps*	Mobile Apps for COVID-19: A Comprehensive Review	(Contribution 18)
